# Evaluation of cytotoxicity features of antimicrobial peptides with potential to control bacterial diseases of citrus

**DOI:** 10.1371/journal.pone.0203451

**Published:** 2018-09-07

**Authors:** Rosangela Naomi Inui Kishi, Dagmar Stach-Machado, Junya de Lacorte Singulani, Claudia Tavares dos Santos, Ana Marisa Fusco-Almeida, Eduardo Maffud Cilli, Juliana Freitas-Astúa, Simone Cristina Picchi, Marcos Antonio Machado

**Affiliations:** 1 Centro de Citricultura Sylvio Moreira, Instituto Agronômico, Cordeirópolis, São Paulo, Brazil; 2 Instituto de Biologia, Universidade Estadual de Campinas, Campinas, São Paulo, Brazil; 3 Faculdade de Ciências Farmacêuticas, Universidade Estadual Paulista Julio de Mesquita Filho, Araraquara, São Paulo, Brazil; 4 Instituto de Química de Araraquara, Departamento de Bioquímica e tecnologia química, Universidade Estadual Paulista Julio de Mesquita Filho, Araraquara, São Paulo, Brazil; 5 Embrapa Cassava and Fruits, Cruz das Almas, Bahia, Brazil; University of San Agustin, PHILIPPINES

## Abstract

Antimicrobial peptides (AMPs) can be found in various organisms, and could be considered an alternative for pesticides used to control plant pathogens, including those affecting citrus. Brazil is the largest producer and exporter of frozen concentrated orange juice in the world. However, the citrus industry has been affected by several diseases such as citrus canker and *huanglongbing* (HLB), caused by the bacteria *Xanthomonas citri* subsp. *citri* (*X*.*citri*) and *Candidatus* Liberibacter asiaticus (CaLas), respectively. In order to control these pathogens, putative AMPs were prospected in databases containing citrus sequences. Furthermore, AMPs already reported in the literature were also used for *in vitro* and *in vivo* assays against *X*.*citri*. Since CaLas cannot be cultivated *in vitro*, surrogates as *Sinorhizobium meliloti* and *Agrobacterium tumefaciens* were used. This study reports the evaluation of six AMPs obtained from different sources, two of them from *Citrus* spp. (citrus-amp1 and citrus-amp2), three from amphibians (Hylin-a1, K^0^-W^6^-Hy-a1 and Ocellatin 4-analogue) and one from porcine (Tritrpticin). Peptides K^0^-W^6^-Hy-a1, Ocellatin 4-analogue, and citrus-amp1 showed bactericidal activity against *X*.*citri* and *S*. *meliloti* and bacteriostatic effect on *A*. *tumefaciens*. These results were confirmed for *X*.*citri in planta*. In addition cytotoxicity evaluations of these molecules were performed. The AMPs that showed the lowest hemolytic activities were Triptrpticin, citrus-amp1 and citrus-amp2. Citrus-amp1 and citrus-amp2 not presented toxicity in experiments using *in vivo* model, *G*. *mellonella* and U87 MG cells. To verify the interaction of these AMPs with bacteria and erythrocyte cell membranes, vesicles mimicking these cells were built. Citrus-amp1 and Tritrpticin exhibited higher affinity to bacterial membranes, while Ocellatin 4-analogue and Hylin-a1 showed higher affinity to erythrocyte membranes; exclude their use in citrus. This work demonstrates an essential alternative, trough AMPs obtained from *Citrus* spp., which can be feasibly used to control bacterial pathogens.

## Introduction

Plant diseases have a great impact on the productivity of several crops, and their control is often accomplished only by excessive use of agrochemicals. The emergence of fungal and bacterial pathogens resistant to the active ingredients of pesticides has been reported. Moreover, environmental damage and problems of acute toxicity to animals and humans may occur [1]. Antimicrobial peptides (AMPs) have been used as a new strategy for the control of plant diseases, due to the need for alternatives with less or no toxicity effect and impact on the environment [2].

Antimicrobial peptides are a group of molecules conserved during evolution that can be found in a wide range of organisms [3,4]. Most of these peptides have similar characteristics: (a) they are cationic with two or more positive charges, (b) contain from ten to fifty amino acid residues, (c) have 50% hydrophobic residues, and (d) form α-amphipathic helix [5,6].

The mode of action of AMPs are not completely elucidated, but most of them inhibit microbial growth by cell membrane permeabilization through the formation of pores or other lytic effects which permit the efflux of ions and essential nutrients to cells [7,8,9]. These AMPs, when reaching critical concentration, are introduced into the cell membrane, causing disintegration of the lipid bilayer by breaking it or forming pores [10].

Additionally, AMPs can act in cell division, macromolecules and cell wall synthesis [11], and interact with lipopolysaccharides (LPS) of the cell wall [12, 13]. A potential problem is that peptides acting on membranes are not completely selective to microbial cells and may present potential toxicity to eukaryotic cells as well [10,14,15]. Hence, detailed studies should be carried out in order to determine whether or not a given AMP is feasible for plant pathogen control. Biophysical studies showed two important factors in the selectivity of membranes by AMPs: (a) electrostatic interaction between the cationic peptide and the fatty acid membrane profile of the bacterium, which is more negatively charged than mammalian cells with about 25% of anionic lipids, and (b) the presence of a large amount of cholesterol in eukaryotic membranes, which stabilize the lipid bilayer and inhibits the disruption of cell membranes [16,17].

Cell membranes have been the subject of many studies due to their importance in cell biology, determining the composition of the membrane structure [18] interacting with membrane proteins [19] and acting in transmembrane translocation [20]. Thus, vesicles that mimic prokaryotic and eukaryotic cell membranes have been used to assess the peptide/membrane interaction, since their lipid bilayer structures are identical to the lipid portion of the cell membranes. Even though the vesicles are a simple system and do not harbor proteins or carbohydrates in their composition [21], they can be very useful in the screening of AMPs to determine affinity to eukaryotic or prokaryotic cells.

In agriculture, antimicrobial peptides have been used in the production of transgenic plants resistant to economically important diseases, such as citrus canker and *huanglongbing* (HLB) [22, 23]. An advantage of this approach is that AMPs can have a broad spectrum of action, in this way the use of a single peptide could confer resistance to multiple plant pathogens [24].

In this study we evaluated six peptides obtained from different sources, two of them from *Citrus* spp., three from amphibians and one from porcine, against bacterial pathogens responsible for severe losses in citrus production worldwide or their surrogates. To verify the toxicity of these peptides we evaluated the hemolytic effect and for citrus peptides we performed a detailed study on the cytotoxicity effects trough toxicity to *Galleria mellonella* and U 87 MG cells. In addition to those parameters, phytotoxicity on citrus tissue was evaluated. Citrus-amp 1 can be considered the most interesting AMP tested, since it showed bactericidal effect on the prokaryotic cells tested, but very little or no effect on the eukaryotic cells. Additionally, it was obtained from the citrus genome, which certainly should reduce deregulation problems and avoid the addition of foreign sequence in citrus. Hence, we propose this AMP could be further investigated for potential use.

## Materials and methods

### Antimicrobial peptides and peptides syntheses

Twenty eight antimicrobial peptides were prospected, twenty two of which were from CitEST (citrus expressed genome) [25] Database, with 110,903 unigenes and six were selected from the complete genomes of *C*. *sinensis* (46,147 proteins) and *C*. *clementina* (35,976 proteins) (www.phytozome.org) through the Kamal software, designed by Brand et al. [26] and the Helical Wheel projections program available at http://rzlab.ucr.edu/scripts/wheel/wheel.cgi. The parameters used for prospecting AMPs were the presence of cationic, positive charge, length of 10 to 50 amino acids; 50% of hydrophobic residues and ability to form amphipathic alpha helix. According to the criteria adopted in the prospection of AMPs, two novel peptides (citrus—amp1 and citrus–amp 2) obtained from citrus sequences from Phytozome were selected. In addition four AMPs already described in the literature (Table 1) were used. Physicochemical properties of the AMPs used in this work were evaluated (Table 2).

**Table 1:** Description of peptides evaluated in this work.

Peptide	Sequence	Source	Reference	
Hylin-a1	IFGAILPLALGALKNLIK	*Hypsiboas albopunctatus*	[5, 27]	
K^0^-W^6^-Hy-a1	KIFGAIWPLALGALKNLIK	Analogue of Hylin-a1	[5]	
Tritrpticin	VRRFPWWWPFLRR	Porcine cathelicidin	[28]	
Ocellatin4-analogue	KLLKFVTKVGKAIFKALIKAI	*Leptodatylus ocellatus* Analogue of Ocellatin 4	[29]	
Citrus-amp1	IETFLKQLRSAANKIVGL	*Citrus sinensis*	This work	
Citrus-amp2	LESLASSAVRTANKARAKL	*Citrus aurantium*	This work	

**Table 2:** Physico-chemical properties of AMPs evaluated.

Properties	AMPs					
	Citrus-amp1	Citrus-amp2	K^0^-W^6^-Hy-a1	Ocellatin4-analogue	Hylin-a1	Tritrpticin
MW	2001.4	1986.3	2066.6	2330	1865.3	1902.2
pI	10.0	11.0	10.3	10.7	10.0	12.48
NR	18	19	19	21	18	13
NC	2	3	3	6	2	4
CR	K(2), R(1), E(1)	K(2), R(2), E(1)	K(3)	K(6)	K(2)	R(4)
H	0.450	0.146	0.754	0.560	0.820	0.819
HM	0.588	0.382	0.518	0.607	0.473	0.411

http://web.expasy.org/cgi-bin/protparam/protparam and http://heliquest.ipmc.cnrs.fr/cgi-bin/ComputParams.py

MW–Molecular weight (g/mol); pI—isoelectric point; NR—number of residues; NC–net charge; CR–charged residue; H–Hydrophobicity; HM–Hydrophobic moment.

The peptides were manually synthesized by solid-phase peptide synthesis using standard 9-fluorenylmethyloxycarbonyl (Fmoc) protocol, according to Vicente et al. [30]. The syntheses were performed at the Department of Biochemistry and Chemical Technology of UNESP—Universidade Estadual Paulista, coordinated by Dr. Eduardo Maffud Cilli.

### Microorganisms and growth conditions

Two important citrus pathogenic bacteria were addressed in our study: *X*.*citri* (isolate 306), the causal agent of citrus canker, and CaLas, causal agent of HLB. Since the latter cannot be cultivated *in vitro*, surrogates phylogenetically related to CaLas [31] were used in the assays. The surrogates chosen were *S*. *meliloti* SEMIA 165 (= USDA 1002) and *A*. *tumefaciens* (GV3101/PMP90), provided by FEPAGRO (Fundação Estadual de Pesquisa Agropecuária) and by Dr. Janete Desidério, UNESP (Universidade Estadual Paulista, Jaboticabal, São Paulo, Brazil). The effect of the AMPs on *Methylobacterium* sp., an endophytic commonly found at citrus in Brazil [32] and kindly provided by Dr. Wellington Luiz Araújo, Instituto de Ciências Biomédicas, Universidade de São Paulo, Brazil, was also assessed. *Methylobacterium* sp. and *X*.*citri* was cultured in NBY solid medium and then in NBY liquid medium according to Amaral et al. [33] for 48 hours at 28°C and 180 rpm. *S*. *meliloti* and *A*. *tumefaciens* were grown in YM medium (Yeast Mannitol Medium) (0.5 g/L KH_2_PO_4_, 0.2 g/L MgSO_4_ 7.H_2_O, 0.1g/L NaCl, 10 g/L mannitol, 0.5 g/L yeast extract, 9g/L agar, pH 6.8) [34] and then in YM liquid medium for 48 hours at 28°C and 180 rpm. In addition to the *in vitro* assays, *X*.*citri*::GFP [35], obtained from Dr. Adrián A. Vojnov (Instituto de Ciencia y Tecnología Dr. Cesar Milstein, Fundación Pablo Cassará, Consejo Nacional de Investigaciones Científicas y Técnicas (CONICET), Buenos Aires / Argentina), was used for the *in vivo* assays.

### Antimicrobial activity *in vitro*

Antimicrobial peptide activity was performed *in vitro* using a microliter plate assay (96 well cell culture cluster—Costar^®^3599). Fifty microliters of inoculum at 10^4^ CFU/mL prepared in PBS buffer (Phosphate buffered saline) was mixed with 50 μL of the peptide diluted in distilled water filter sterilized (dH_2_O). The final concentration of peptide evaluated ranged from 1–64 μM, and hylin-a1 and PBS were used as positive and negative control for AMP, respectively. The minimal inhibitory concentration (MIC) was the concentration that did not allow any development of the bacteria, assessed by a microplate reader model 3550 Bio-Rad^®^ at 595nm. Three replicates were prepared for each concentration evaluated.

At the end of the growth incubation, minimum bactericidal concentration (MBC) for each organism was determined by serial dilution using the microdrop technique, as described elsewhere [36]. The Petri dishes were maintained at 28°C for three days to obtain CFU/mL.

### Hemolysis assay

The methodology used for hemolytic assay was based on Onuma et al. [37]. Fresh human red blood cells (RBC) (O +) were washed three times with saline buffer (2.76 g/L de NaH_2_PO_4_ and 9 g/L NaCl, pH 7.4). A suspension of erythrocytes 1% v/v and peptides (at 25, 50, 75 and 100 mM) were prepared with the Tris-saline buffer. Aliquots of 100 μL blood suspension and 100 μL peptide solution were incubated at 37°C for 1 hour. The samples were centrifuged at 3000 x g for 2 min. Aliquots of 100 μL were added to the wells of “96-well cell culture cluster—Costar^®^3599” and the absorbance was determined at 405nm in a microplate reader model 3550 Bio-Rad^®^_._ As a positive control for hemolysis (100% lysis), 1% (v/v) Triton X-100 solution was used. PBS buffer was used as a negative control (0% lysis). The assay was performed in triplicate to determine the percentage of hemolysis at different concentrations of peptides. High concentrations of peptide were used for all cytotoxicity assays with the objective to verify the possible effect of these AMP against mammalian cells in extreme situation. Hemolysis percentage was calculated as follows:

$$\%\;Hemolysis=\left[\frac{\left(Abs\;405\;nm\;of\;AMP\;Solution-Abs\;405\;nm\;of\;PBS\right)}{\left(Abs\;405\;nm\;TritonX\;100-Abs\;405\;nm\;of\;PBS\right)}\right]x100$$

### Evaluation of AMPs in plants

Plants of *Citrus sinensis* (L.) Osbeck grafted on *Citrus limonia* Osbeck were grown in pots of 5 liters with “Terra do sol Multplant” citrus substrate. After one year, these plants were pruned and leaves with approximately 45 days were used for the assay. The experiments with *X*.*citri* were conducted in an A2 quarantine greenhouse at Instituto Agronômico de Campinas (IAC). The plants were irrigated and fertilized accordingly, and the average temperature of the greenhouse was 30°C.

Two independent experiments with one plant were performed. One hundred microliters of a suspension composed of 50/50 (v/v) of AMP and *X*.*citri* (10^4^ CFU/mL) as described for the *in vitro* evaluation, was inoculated in the abaxial leaf. The replicates were consisted of three branches per plant and in each branch three leaves of similar age were inoculated at three different points by leaf, being in total 27 inoculations by plant. PBS buffer and hylin-a1 were used as negative control and positive control, respectively. After fourteen and twenty-one days after inoculation, one leaf by each branch was collected for qualitative and quantitative evaluations. Qualitative evaluation was done visually through the fluorescence of *X*.*citri*::GFP (positive control). Isolation and serial dilutions were performed from the average of nine inoculations points of citrus tissues [36].

### Phytotoxicity assessment

For phytotoxicity assays in plants, 10 μL of each peptide at 128 and 64 μg/mL were infiltrated in detached leaves of sweet orange by syringe infiltration according to Makovitzki et al. [38]. Hylin-a1 and PBS Buffer were used as positive and negative control. In this assay, two independent experiment was performed in which three leaves was inoculated in the abaxial leaf at three different points and qualitative evaluation was made to verify the possible toxicity of AMP. The leaves were put on agar-plates (1%) for 7 days and maintained on controlled conditions at 26°C and photoperiod of 16h:8h (16 hours of light: 8h of dark).

### Toxicity in *Galleria mellonella* larvae

The peptides citrus–amp1 and citrus–amp2 were selected for toxicity assay in *G*. *mellonella* [39]. Larvae with 0.15 to 0.20 g were randomly selected for the experiments and were kept without food in Petri plates at 37°C in the dark for 24 h prior to use. Each larva was injected with 10 μL of peptides at 64 and 128 μM into the last proleg using a Hamilton micro-syringe. The concentration was selected according to the MIC (64 μM), and to confirm the low toxicity we used the double concentration (128 μM). The larvae were incubated at 37°C and survival was evaluated daily for 7 days. Group of 8–9 larvae was used to each concentration of peptides and 10 μL of PBS was injected in the control group. Two independent experiments were performed.

### Cytotoxicity in U87 MG cells

U87 MG, a human Glioblastoma cell line frequently used as a model for cytotoxicity evaluation was adopted in our study for the peptides citrus–amp1 and citrus–amp2. Cells were plated in 96 wells plate (5x10^4^ cells per well), incubated overnight and maintained in a humidified atmosphere at 37°C and 5% CO_2_. After the incubation period, cells were treated with peptides varying from 1μM to 128 μM and then incubated for 24h. Untreated and cells treated with DMSO (dimethyl sulfoxide) were used as positive and negative control for the viability of U87 MG cells. Six replicates were done by each concentration.

Cytotoxicity was performed using alamarBlue® cell viability reagent [40], a cell health indicator, through the reduction of viability of living cells. Viable cells convert Resazurin (blue) to resorufin, a compound that emits red fluorescent light. Alamar Blue medium was prepared by mixing medium with alamarBlue solution (Thermo Ficher Scientific) in a ratio of 10:1. After treatment with the peptides for 24 h, the medium was discarded, and Alamar Blue medium (100 μL) was transferred to a 96-well microplate. The microplates were incubated with Alamar Blue at 37°C for 4 h, and absorbance was measured using Epoch2 Microplate Reader (Biotek) in 570 nm. Cell viability was calculated using the ratio of the absorbance between treated and untreated cells and the data were expressed as percentages. Three independent experiments were performed.

### Vesicle permeabilization assay

Two kinds of large unilamellar vesicles (LUVs) were made according to Lorenzón et al. (2012) [21]. One LUV has the main lipid of eukaryotic cells—100% 1-Palmitoyl-2-Oleoyl-*s*n-Glycero-3-Phosphocoline (DPPC). The other contains 80% DPPC and 20% 1-Palmitoyl-2-Oleoyl-*s*n-Glycero-3-[Phospho-rac-(1-glycerol)] Sodium Salt) (POPG). This last had negative charge, and was used to mimic bacteria cell membrane (Avanti Polar lipids). Both LUVs were prepared by adding the appropriate amounts of lipids in 4:1 chloroform-methanol mixture in a round-bottom flask. The solvent was evaporated using nitrogen gas until obtaining a lipid biofilm that was placed under a vacuum chamber overnight and hydrated with 80 mmol/L of 5 (6)-carboxyfluorescein (CF) (Sigma^®^ Life Science) in Tris (0.01 mol/L, pH 7.4) and NaCl (0.15 mol/L), at 60°C to a final lipid concentration of 15 mmol/L. This suspension was extruded 40 times through Nucleopore polycarbonate filters (100 nm pore size) using an extruder system from Avanti Polar Lipids at approximately 40°C. Vesicles were separated from non-encapsulated CF by gel filtration on a Sephadex G-50 (GE Healthcare Life Sciences) column using Tris-NaCl buffer for elution. The release of CF from vesicles was measured by the fluorescence intensity at a wavelength of 520 nm (492-nm excitation wavelength) after the addition of the peptides. Determination of the value for 100% of release was achieved using Triton X-100. Data were obtained using a fluorescence spectrophotometer (Cary Eclipse; Varian). The experiments were performed at 25°C. The final values were calculated as the percentage of CF release after the addition of peptide into the system, obtained using the equation percent release: [(*F_fin_* − *F_in_*)/(*F_max_* − *F_in_*)]*x*100, where F_in_ and F_fin_ represent the initial and final fluorescence values before and after the addition of peptides, respectively. F_max_ represents the fluorescence after the addition of Triton-X. The concentration of peptides used was the one that could detect CF release and consequently pore formation. All experiments were performed in duplicate, and the mean was analyzed by Tukey Test (*P*<0.05) through ASSISTAT [41].

## Results and discussion

### Selection of AMPs

**Fig 1:**
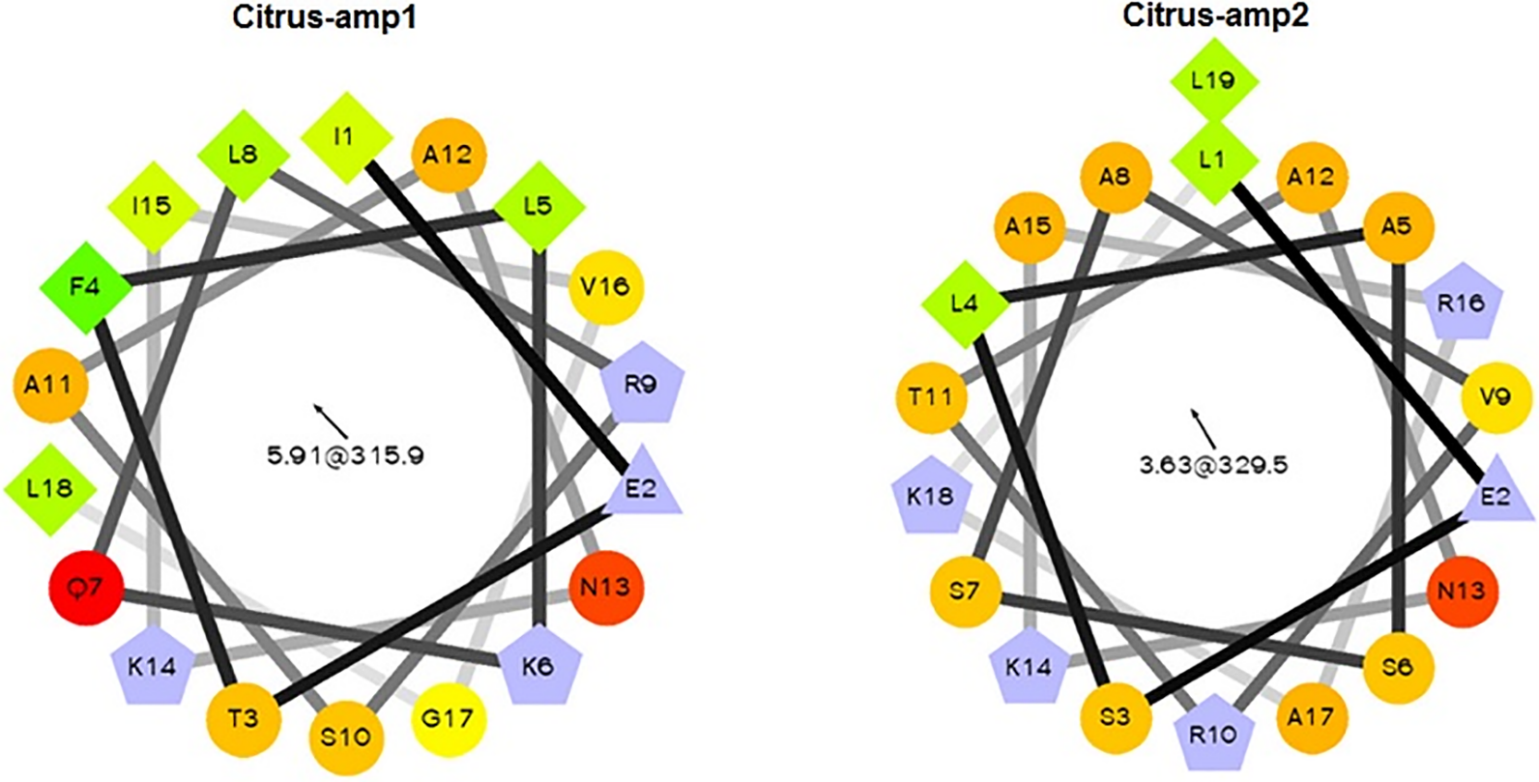
Helical wheel projections of AMP (Hydrophilic residues as circles, hydrophobic residues as diamonds, and potentially positively charged as pentagons). Hydrophobicity degree: the most hydrophobic residue is green and low hydrophobicity is coded as yellow. Hydrophilic residues are coded red with pure red being the most hydrophilic (uncharged) residue, and the degree of red color represent the level of hydrophilicity. Light blue represents charged residues.

Through the criteria used for prospecting putative AMPs in citrus sequences, peptides obtained from hypothetical protein of *Citrus sinensis* (citrus–amp1) and Pyruvate Kinase protein (citrus-amp2) from *Citrus aurantium* were chosen. The structures and sequence of these peptides were refined according the ability to form amphipathic α-helix. In Fig 1, the amphipathic structures for both peptides are illustrated.

### Antimicrobial and hemolysis activities

The antimicrobial and hemolytic activities were determined to evaluate the effect of these AMPs against bacterial growth and eukaryotic cells. Table 3 presents the antimicrobial activity expressed in MIC (minimal inhibitory concentration) and MBC (minimal bactericidal concentration).

**Table 3:** Minimal inhibitory concentrations (MICs) and minimal bactericidal concentrations (MBCs) against microorganisms and hemolysis activity percentage of peptides.

AMP	MIC (μM)				MBC (μM)^1^				Hemolysis activity (%)			
	*X*.*ciri*	*S*. *meliloti*	*Met* sp.	*A*. *t*	*X*.*ciri*	*S*. *meliloti*	*Met* sp.	*A*.*t*	25 mM	50 mM	75 mM	100 mM
Citrus-amp1	32	64	64	64	32	64	64	ND*	1	0	0	1
Citrus-amp2	ND	64	ND	ND	ND	64	ND	ND	1	0	0	0
K^0^-W^6^-Hy-a1	2	4	4	ND	2	62	8	ND*	14	53	83	99
Ocellatin4-Analogue^1^	3	3	3	3	3	14	7	55*	88	100	100	100
Tritrpticin	8	4	ND	ND	8	8	ND	ND	1	3	10	28
Oxytocin^2^	-	-	-	-	-	-	-	-	0	0	0	1
PBS^3^	-	-	-	-	-	-	-	-	-	-	-	-
Hylin-a1^4^	4	ND	9	17	9	ND	17	17	-	-	-	-

*X*.*citri*–*Xanthomonas citri* subsp. *citri*; *S*.*meliloti–Sinorhizobium meliloti*; *Met* sp.–*Methylobacterium* sp.; *A*.*t–Agrobacterium tumefaciens;* ND: Not determined (higher than the concentrations evaluated).

*Growth reduction

^1^Positive control for hemolysis

^**2**^Negative control for hemolysis activity

^3^Negative control for MIC/MBC

^4^ Positive control for MIC/MBC.

The two peptides isolated from citrus did not cause hemolysis in red blood cells (RBC) (Table 3). In fact, citrus-amp2 did not show any antimicrobial activity, probably due to the weak hydrophobicity face and consequently low amphipathic structure presented (Fig 1). On the other hand, citrus-amp1 can be considered a promising antimicrobial peptide, which may be able to control important citrus pathogens. The main features that favor the interaction with bacteria membranes are at least 50% of hydrophobic residues and regular amphipathic structure. This AMP was effective to control *X*.*citri* (MIC = MBC of 32 μM) and *S*. *meliloti* (MIC = MBC of 64 μM) (Table 3), but not *A*. *tumefaciens*. This surrogate showed MIC of 64 μM (Table 3), which was the highest dosage tested. Even at such high dosage, citrus-amp1 was not able to kill the bacteria, only to exhibit a growth reduction of 10^3^ CFU/mL.

The four non citrus AMPS was selected based on their effects previously observed by other works [5,28,29,42]. Moreover the peptide hylin-a1 was selected to be our positive control on antimicrobial activity and in hemolytic activity. The peptide K^0^-W^6^-Hy-a1 (analogue to hylin-a1) [5] was selected to verify if the difference with hylin-a1 can interfere in antimicrobial activity in our study. As described by Crusca et al. [5] hylin-a1 has strong antimicrobial and hemolytic activities, and was used as a positive control for MIC and MBC determination. However, this peptide did not suppress the growth of *S*. *meliloti*, even at the highest concentration tested of 64 μM (Table 3). On the other hand, this AMP was very efficient to control *X*.*citri*, but due to the high hemolytic activity (Table 3), it is considered inappropriate for use, due to the risk to the health of citrus consumers. As showed by Crusca et al. [5], the addition of Lysine residue in N-terminus and substitution of Leucine by tryptophan amino acid at position 6 (K^0^-W^6^-Hy-a1) improved the biological activity. The insertion of this residue at hydrophobic face of amphipathic α-helix may increase the cytotoxicity [42] and hemolysis. In our study, we used in hemolytic activity studies higher dosages of peptide (the IC_50_ found previously to this peptide was 4 μmol/L) with the purpose to assurance the effect in extreme situations. The maximum dosage tested of 100 mM caused more than 99% of cell lysis (Table 3). K^0^-W^6^-Hy-a1 was effective against *X*.*citri* (MIC = MBC = 2 μM) and *Methylobacterium* sp. (MIC = 4 μM and MBC = 8 μM). This AMP was less efficient in controlling the bacterial surrogates of CaLas, *S*. *meliloti* (MBC of 62 μM, but with detectable growth reduction at 31 μM of 10^4^ CFU/mL and 10^2^ CFU/mL for the concentrations ranging from 4 to 15 μM). For *A*. *tumefaciens*, it was not possible to determine MBC, but a growth reduction of 10^3^ CFU/mL was observed from 4 to 62 μM.

Ocellatin4 (GLLDFVTGVGKDIFAQLIKQI-NH(2)) is a cytolytic peptide obtained from skin secretion of the South American frog *Leptodactylus ocellatus* [29]. The analogue of this peptide named as, Ocellatin4-analogue (KLLKFVTKVGKAIFKALIKA**I**) [43] has positive charge at its N-terminus (lysine residue), similarly to K^0^-W^6^-Hy-a1. This peptide showed the same MIC values for *X*. *citri*, *S*. *meliloti*, *Methylobacterium* sp. and *A*. *tumefaciens*, and high hemolytic activity. Nascimento et al. [29] reported activity of ocellatin 4 at 14.3 mmol/L on human erythrocytes and suggested that the interaction between membranes of mammals and ocellatin 4 is favored by neutral charge of peptides at pH 7.0. Ocellatin4-analogue has higher positive charge, which could promote higher hemolytic activity (88% with 25 mmol/L of peptide). On the other hand, only 3 μM of the peptide was enough to control the growth of the bacteria, which is a much lower concentration than that needed to cause hemolysis (Table 3). Hence, the use of this peptide to control *X*.*citri* might be feasible from the scientific perspective, although it would have serious difficulties for deregulation and most certainly its use in agriculture would not be allowed. Additionally, it should be avoided due to the presence of two phenylalanine residues that can improve the cytotoxicity activity of this peptide. In this case, the presence of the above mentioned residues and the strong hydrophobic face (Fig 1) may increase the hemolytic activity. The MBC observed for *X*.*citri*, *S*. *meliloti* and *Methylobacterium* sp. were 3 and 14 μM and 3 and 7 μM, respectively. For *A*. *tumefaciens* the MIC and MBC concentrations were 3 and 55 μM, respectively. At the MIC concentration, the reduction in *A*. *tumefaciens* growth was of only 10^2^ CFU/mL when compared to the positive control (bacteria only).

The peptide Tritrpticin is a Trp-rich cationic antimicrobial peptide derived from a porcine cathelicidin and also has a broad spectrum of antibacterial, antifungal, and hemolytic activities. This peptide is composed of high percentages of arginine (30%), tryptophan (23%) and proline (15%), which make it unique. It has a good potential to control Gram-negative and Gram-positive bacteria, as well as some fungi [28]. In this work, this peptide was able to control *X*.*citri* and *S*. *meliloti*, and the hemolytic activity was effective only at concentrations much higher than those required for antimicrobial activity (Table 3), a feature also observed and described by Yang et al. [28]. These authors observed that the peptide showed more activity against Gram-positive than Gram-negative bacteria. In this work, this peptide did not show any effect on the endophyte *Methylobacterium* sp., nor on *A*. *tumefaciens*, often used for plant transformation. Thus, whether or not it could be used to control CaLas remains to be proven, but it seems interesting for the management of citrus canker and could be considered for the citrus breeding program using Agrobacterium-mediated transformation (Table 3).

Antimicrobial peptides have shown higher instability to proteolytic enzymes in human saliva and gastric fluids. Na et al. [44] verified by HPLC that Antimicrobial Decapeptide (KSL) was degraded by both. The degradation by saliva was dependent on the concentration of KSL, and in the absence of enzymes in the saliva, the degradation was not observed for over 24 h. Despite these results, it is possible that AMPs are not fully degraded by saliva or gastric fluid and should be toxic to mammals. Therefore, we estimated some toxicity parameters in this work with the purpose to detect the possible damages that could occur to eukaryotic cells. Additional evaluation should be performed before the release of any product to the consumers.

The hydrophobic moment (HM) (Table 2) is highly correlated with hemolysis. Higher HM can result in significant increase in membrane permeabilization and hemolytic activity [3]. In this work, we observed that the Ocellatin4 –analogue, which presents highest HM among the AMPs evaluated, showed high hemolytic activity and high permeabilization of mimic vesicles for mammal cells, as observed in vesicle permeabilization assay.

The *in vitro* assays are important to determine the potential of AMPs against plant pathogens, although experiments in plants should be conducted to verify the effect of these in natural conditions. Thus, additional experiments with transgenic plants should be performed to observe the real potential against CaLas and not only its surrogates. To evaluate the possible effects of these AMPs in plants for citrus canker, a suspension with AMPs and *X*.*citri*::GFP was used in plants of *C*. *sinensis*, as describe below.

### Effect of AMPs in plants

**Fig 2:**
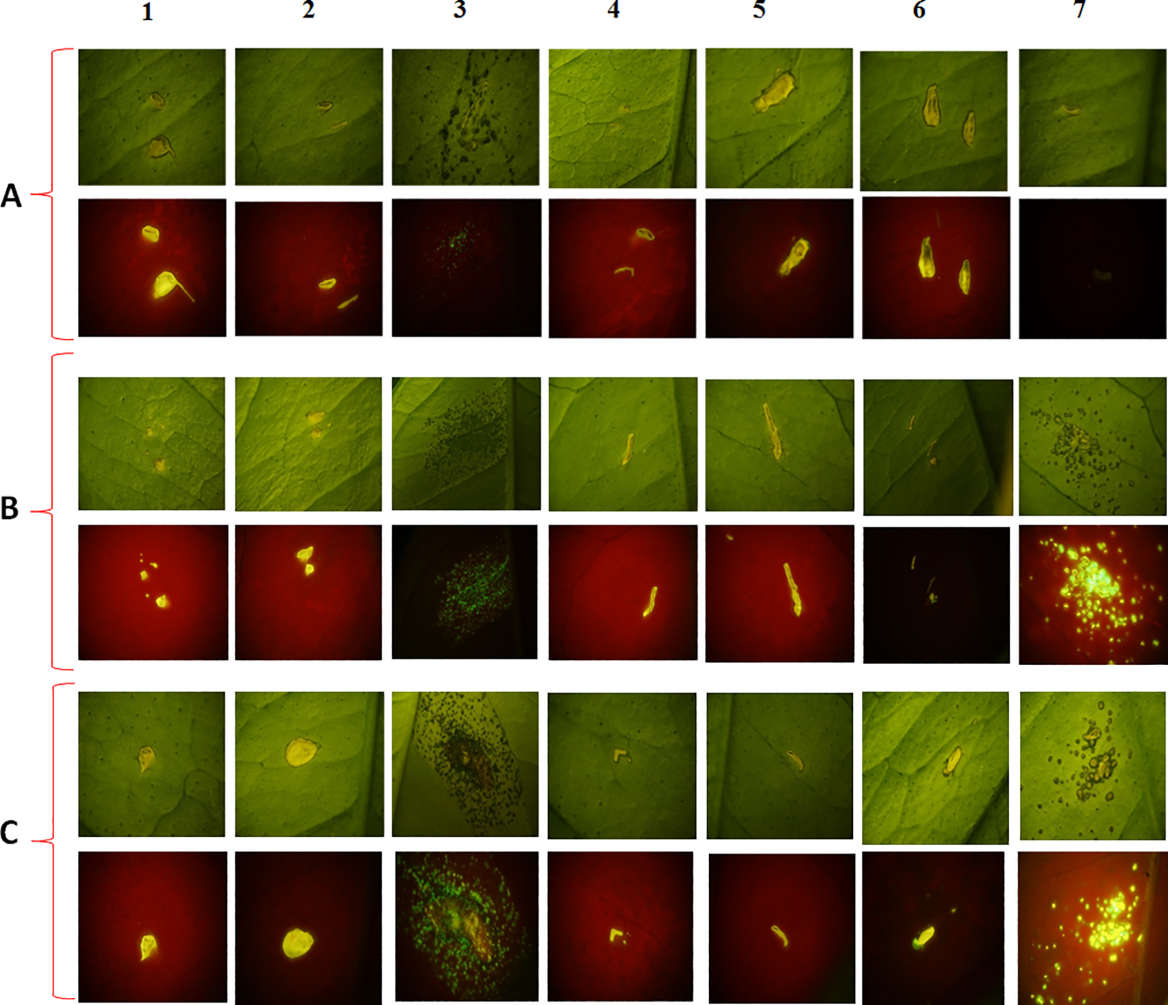
Qualitative evaluation of AMP activity against *X*.*citri* in *C*. *sinensis*. (A) 7dpi, (B) 14 dpi, (C) 21dpi, (1) Tritrpticin, (2) PBS buffer, (3) *X*.*citri*::GFP, (4) Hylin-a1, (5) Ocellatin4-Analogue (6) Citrus–amp 1 and (7) K^0^-W^6^-Hy-a1.

Citrus canker evaluation was carried on at 7, 14 and 21 days post inoculation (dpi). At the first seven days, only the positive controls (*X*.*citri*::GFP) showed canker symptoms and supported bacterial growth by qualitative evaluation (Fig 2). During the following periods of evaluation, at 14 and 21 dpi, both the symptoms and the presence of *X*.*citri*::GFP were detected for citrus-amp1 and K^0^-W^6^-Hy-a1. The symptoms observed on the leaves treated with these AMPs were less severe than those observed in the positive control (Fig 2). However, after quantitative analysis through serial dilutions (S1 Table), it was possible to obtain around 10^8^ CFU/mL for *X*.*citri*::GFP, 10^7^ CFU/mL for K^0^-W^6^-Hy-a1 and for citrus-amp1 (Table 4). The bacterial concentration did not change between 14 and 21 dpi for all peptides evaluated (Table 4). The peptides citrus-amp1 and K^0^-W^6^-Hy-a1 showed good control under *in vitro* conditions; however, the metabolites present in the plants, stability of AMPs for protease and favorable conditions to *X*.*citri* in plants may have affected the activity of the AMPs. According to Bessale et al. [45], the stability of AMPs to protease degradation is an important feature to determine the half-life of the peptide under natural or uncontrolled conditions, as in greenhouse and field experiments. To verify degradation by proteases, some enzymes such as trypsin, chymotrypsin and pronase can be added to the peptides and degradation can be monitored by HPLC. In order to increase the stability of the AMPs, peptide analogues can be developed by the incorporation of D-amino acids, since few enzymes recognize the amide bonds involving D-amino acids. This strategy may promote an increase in antimicrobial activity, and reduction in proteolytic and hemolytic activities [46].

**Table 4:** Isolation of *X*.*citri* at 14 and 21 dpi from leaves of *C*. *sinensis*.

AMP	CFU/mL	
	14dpi	21dpi
Ocellatin4-Analogue	0	0
Hylin-a1*	0	0
Tritrpticin	0	0
K^0^-W^6^-Hy-a1	4.7 x 10^6^ ± 0.52	2.0 x 10^7^ ± 1.90
Citrus-amp1	9.6 x 10^6^ ± 0.75	3.5 x 10^7^ ±0.84
*X*.*citri*::GFP	1.5 x 10^8^ ± 4.4	4.5 x10^8^ ± 1,38

*Hylin-a1 –positive control for AMP

Data expressed as mean±SD

For the peptides Ocellatin4-analogue, Hylin-a1 and tritrpticin neither canker symptoms nor *X*.*citri*::GFP growth were observed (Fig 2). However, as previously shown, Ocellatin 4-analogue and Hylin-a1 exhibited the highest hemolytic activity among the peptides evaluated (Table 3) and the use of tritrpticin, AMP from porcine origin could be a problem for the consumption in some countries.

In this work we observed some activity of AMPs against *X*.*citri in vitro* and *in planta*. Moreover, these results demonstrate the potential use of these AMPs for transgenic plants. Hao et al. [22] described endogenous citrus thionins peptide with the purpose to obtain citrus resistant to HLB and *X*.*citri*. The transgenic plants developed by these authors were challenged by *X*.*citri* 3213 and they obtained a considerable reduction in canker symptoms and bacterial growth. They also evaluated the response of these plants to HLB and observed a reduction in CaLas titer. Stover et al. [24] also tested AMPs from several sources *in vitro* against *X*.*citri* and CaLas surrogates. Moreover, there are other studies which demonstrated that the expression of interesting genes as dermaseptin [47], sarcotaxin IA [48] and plant thionin peptide [23] in citrus plants can provide considerable canker resistance.

### Evaluation of phytotoxicity of AMPs

**Fig 3:**
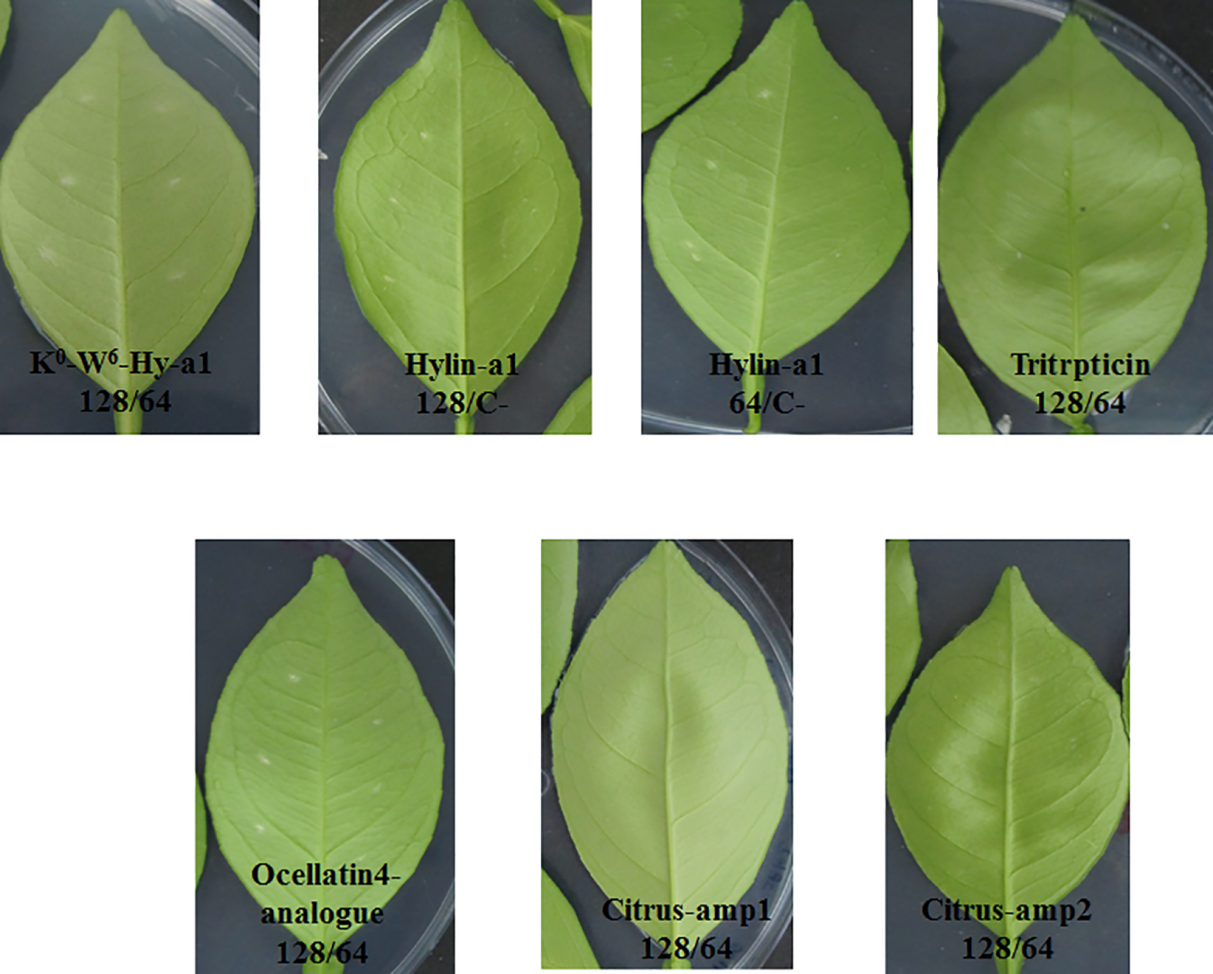
Phytotoxicity of peptides K^0^-W^6^-Hy-a1, Hylin-a1 (positive control), C- negative control, Tritrpticin, Ocellatin 4-analogue, citrus-amp1 and citrus-amp2 at 128 and 64 μg/mL.

The peptide Hylin-1 and PBS buffer were used as positive and negative controls of the phytotoxicity assay, respectively. At seven days post inoculation (7 dpi), visual evaluation was performed in detached leaves. No necrosis was observed for any of the peptides evaluated, even for Hylin-a1 used as a positive control at MIC and MBC determination. Makovitzki et al. [38] assessed the toxicity of peptide C14-KLLK in tobacco leaves. They used mellittin as positive control and observed that leaves started to dry out 12 hours post-inoculation (hpi) and exhibited necrosis 24 hpi at the inoculation point. The peptides evaluated did not show any symptoms at 7 dpi (Fig 3). Consequently, for this parameter, all peptides could be considered suitable for use.

### Toxicity of Citrus peptides in *G*.*mellonella*

The toxicity of peptides was also evaluated in *G*. *mellonella*. This is an insect from the order Lepidoptera and is known as greater wax moth. Recently *G*. *mellonella* has been used as a useful *in vivo* model to evaluate toxicity of chemicals, including antimicrobial agents [49, 50, 51]. The simple and inexpensive maintenance of the larvae in laboratory and the fact that the experiments are not subject to ethical considerations make the model very attractive. Additionally, it is possible administer exact amounts of compounds into the larvae and the effect on survival and melanization on them are easily observed [52]. In our study, both citrus–amp1 and citrus–amp2 at 64 and 128 μM (Table 5) were not toxic for this model since the survival percentages of larvae were high (>87%) and similar to the control group (PBS) (S2 Table).

**Table 5:** Survival percentage of *Galleria mellonella* larvae treated with peptides.

AMP	% survival
Citrus-amp1 64μM	88.89 ± 0
Citrus-amp1 128μM	94.50 ± 7.85
Citrus-amp2 64μM	87.50 ± 17.68
Citrus-amp2 128μM	93.75 ± 8.84
PBS (Control group)	96.30 ± 6.41

Data expressed as mean±SD

### Toxicity of citrus peptides in U87 MG cell line

**Fig 4:**
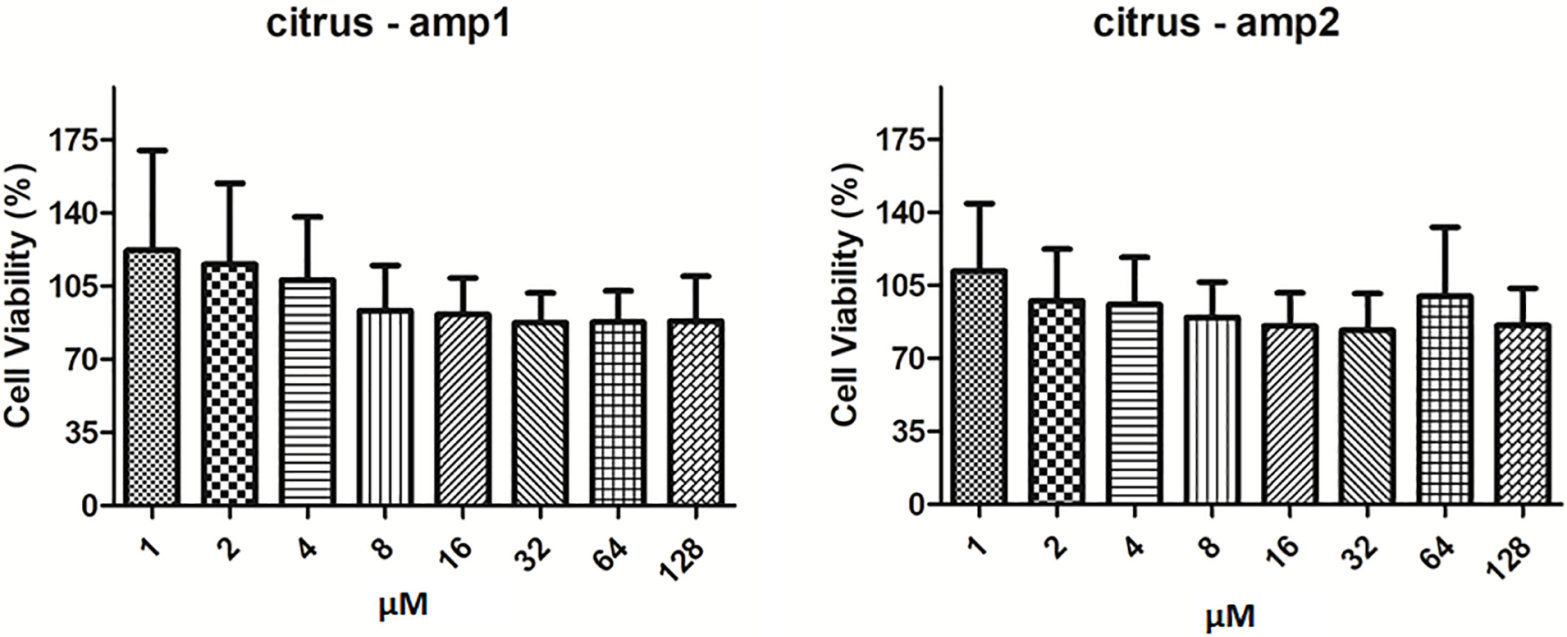
Cell Viability percentage of U87 MG cells treated with peptides. Data expressed as mean±SD.

Cytotoxicity of peptides citrus–amp1 and citrus–amp2 was evaluated in U87 MG cell line [53], a human Glioblastoma (S3 Table). Alamar blue assay was employed to assess mitochondrial ability to reduce resazurin into the fluorescent product resorufin [40]. This method is largely used and well correlated with those obtained from an automatic cell counter when compounds do not induce cellular and nuclear hypertrophy [54, 55]. Fig 4 shows that peptides had low cytotoxicity effects in concentrations of 1–128 μM in this model since the viability percentages of cells were >70% and similar to the control group.

### Vesicles permeabilization

For a better understanding on the interaction between each peptide with bacterial and erythrocytes membranes, permeabilization studies with synthetic vesicles were performed by measuring the release of carboxyfluorescein (CF).

The interaction between peptides and bacteria was evaluated using the vesicles containing POPC (1-Palmitoyl-2-Oleoyl-*s*n-Glycero-3-Phosphocoline)/ POPG 1-Palmitoyl-2-Oleoyl-*s*n-Glycero-3- [Phospho-rac-(1-glycerol)] Sodium Salt) (80%/20%) and POPC (100%). The lipids composition was chosen according to the composition of the outer face of the cell membranes of mammals (basically composed of dipolar phospholipids, like POPC), and bacteria (large quantity of negative phospholipids in two lipid monolayers, or around 80% POPC and 20% POPG).

Ocellatin4-analogue and Hylin-a1 showed increased permeabilization activity of POPC than POPC/POPG vesicles, which suggests that these peptides interact more efficiently with mammal cells than bacterial cells (Table 6). This preference of Ocellatin4-analogue and Hylin-a1 could be related to hydrophobicity, which is an essential characteristic for the interaction between peptide and membrane. Moreover the increase of hydrophobicity is highly correlated with toxicity to mammals cells and loss of antimicrobial specifity [3].

**Table 6:** Permeabilization of vesicles by AMP.

AMP	Concentration (μM)	Permeability Percentage	
		80%POPC and 20%POPG	100%POPC
Citrus-amp1	4.00	45.0 a	3.3 b
Tritrpticin	0.26	33.8 a	7.8 b
Ocellatin4-analogue	0.86	32.8 a	93.9 b
Hylin-a1	0.02	34.4 a	63.3 b
K^0^-W^6^-Hy-a1	0.01	16.8 a	10.5 a

Maximum permeabilization obtained for two different vesicle compositions. Statically difference compared the maximum permeabilization obtained for two different vesicle composition (*P*<0.05 by ASSISTAT). The letters a and b indicated statistically differences in permeabilization according the composition of the vesicles.

The AMP that would probably cause pores indistinctly in mammals and bacterial membranes is K^0^-W^6^-Hy-a1. This unspecific activity presented by K^0^-W^6^-Hy-a1 at vesicles assays, could be correlated with the higher hydrophobicity observed for this peptide in this work (H = 0.754) (Table 2). Higher H can improve hemolysis activity and loss of antimicrobial specificity [3].

For all of the other peptides, significant statistical differences were detected between their activities in the two types of vesicles by the Tukey Test at *P*<0.05 (Table 6). Crusca et al. [5] determined that K^0^-W^6^-Hy-a1 would cause damage to the erythrocyte membrane at 4 μmol/L with the vesicle composition containing 5% DPPA (1,2-dipalmitoyl-3-phosphatidic acid), 47.5% DPPC (1,2-dipalmitoyl-3-phosphocholine), and 47.5% SM (Sphingomyelin). In our study, the vesicle permeabilization occurred at 0.01 μM. The permeabilization caused by K^0^-W^6^-Hy-a1 did not show significant difference between the vesicles evaluated. Thus, it would likely never be deregulated or approved for field application.

The peptides citrus-amp1 and tritrpticin showed increased activity in the membranes mimicking the bacterial cells and did not cause pores in those that mimic the erythrocyte cells (Table 6).

## Conclusions

Many research groups have evaluated the use of AMPs from various sources for the control of plant pathogens, including *X*.*citri*. Some peptides have been selected as putative bacterial control agents and have been used for citrus transformation with that purpose. However, very few–if any–of those studies have addressed possible side effects in using those AMPs, such as hemolysis activity, evaluation of toxicity of chemicals trough the *in vivo* model, *G*. *mellonella*, and using a U87 MG, a human Glioblastoma cell line.

While several of the peptides tested in this study were effective to control the citrus pathogen *Xanthomonas citri* subsp. *citri* (*X*.*citri*), most of them carried undesirable activities against mammal cells and, therefore, are not viable for actual use. Only two of the peptides tested, tritrpticin and citrus-amp1, seem promising due to their antimicrobial and hemolytic evaluations, however the origin of tritrpticin peptide is porcine and this should be considered because there are certain regions of the world where the consumption of products with porcine origin may be unacceptable. In addition, the peptide citrus-amp1, isolated from citrus, can be considered promising for further studies and may be feasible to use in disease management. Besides the antimicrobial activity and no hemolysis effects, this peptide presented low toxicity effects when evaluated in *G*. *melonella* and in U87 MG cell line. Moreover, the use of this peptide could be interesting for the production of cisgenic citrus plants to citrus canker, which could result in greater acceptance by citrus consumers. Additionally, to our knowledge, this is the first report of an AMP obtained from citrus sequences that has an effect on pathogens of that crop.

## Supporting information

S1 Table Detailed information about the effect of AMPs against *X*. *citri* in citrus plants. (XLSX)

S2 Table Toxicity of citrus peptides in *G*.*melonella*. (XLSX)

S3 Table Data from the cell viability U87 MG cells after the treatment with citrus—amp1 and citrus—amp2. (XLSX)
